# Case report: Endoscopic full-thickness resection of gastric metastatic tumor from renal cell carcinoma

**DOI:** 10.3389/fonc.2024.1394784

**Published:** 2024-06-12

**Authors:** Xiaochen Yan, Lina Liu, Wenhao Wang, Chunyan Liu, Zhenqin Cui

**Affiliations:** ^1^ Shengli Oilfield Central Hospital, Affiliated Binzhou Medical University, Dongying, Shandong, China; ^2^ Department of Gastroenterology, Shengli Oilfield Central Hospital, Dongying, Shandong, China

**Keywords:** renal cell carcinoma, gastric metastasis, upper gastrointestinal bleeding, melena, endoscopic full-thickness resection

## Abstract

Renal cell carcinoma (RCC) is a common malignant kidney tumor; however, gastric metastasis is rare. We report the case of an 82-year-old male patient who developed gastric metastasis 12 years after an initial diagnosis of RCC. The patient underwent endoscopic full-thickness resection (EFTR), and the gastric metastatic focus was successfully removed. Postoperative pathology and immunohistochemistry showed that the gastric metastasis originated from RCC. Although gastric metastasis of RCC is rare, it should be suspected in patients with a history of RCC or gastrointestinal symptoms. EFTR is associated with reduced trauma and greater retention of gastric tissue and function. It is a more appropriate choice than surgical resection; however, it requires more endoscopists.

## 1 Introduction

Renal cell carcinoma (RCC) is a common malignant tumor of the kidney (1). It commonly metastasizes to the lungs, bones, liver, brain, and skin. Gastric metastasis is rare and accounts for only 0.2% of cell carcinoma cases (2). The treatment for gastric metastasis of RCC includes surgical resection (total or subtotal gastrectomy), epinephrine injection, and embolization (3, 4).

Here, we report the case of a patient with gastric RCC metastasis. After full communication with the patient and their family, the patient underwent endoscopic full-thickness resection (EFTR), which successfully removed the gastric metastasis. Postoperative pathology and immunohistochemistry confirmed that the gastric metastases originated from the RCC.

## 2 Case description

**Figure 1:**
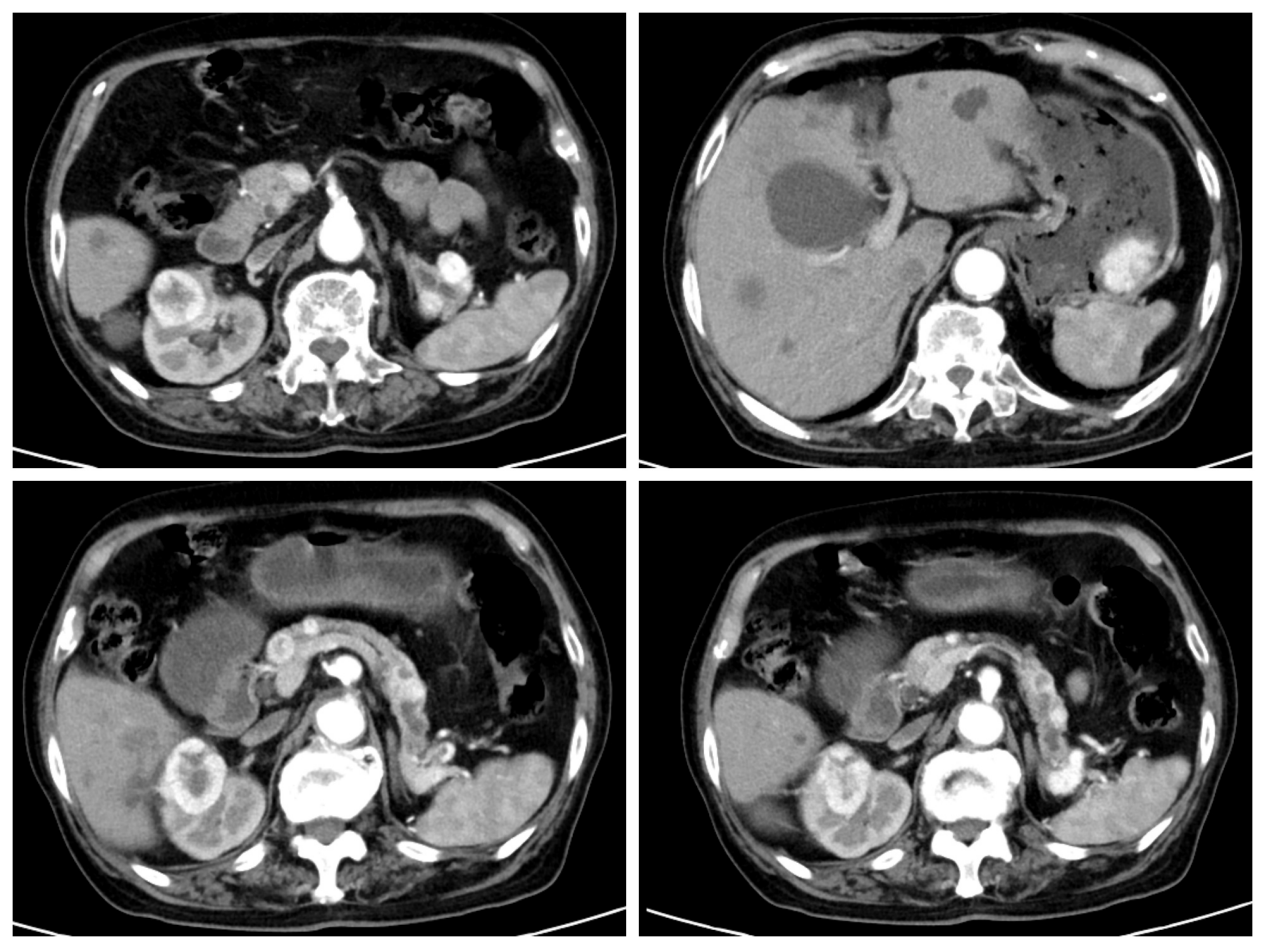
Enhanced CT showing right kidney mass, large curved cauliflower-like mass in the gastric body, multiple enhanced nodules in the pancreas, and splenic hilum.

**Figure 2:**
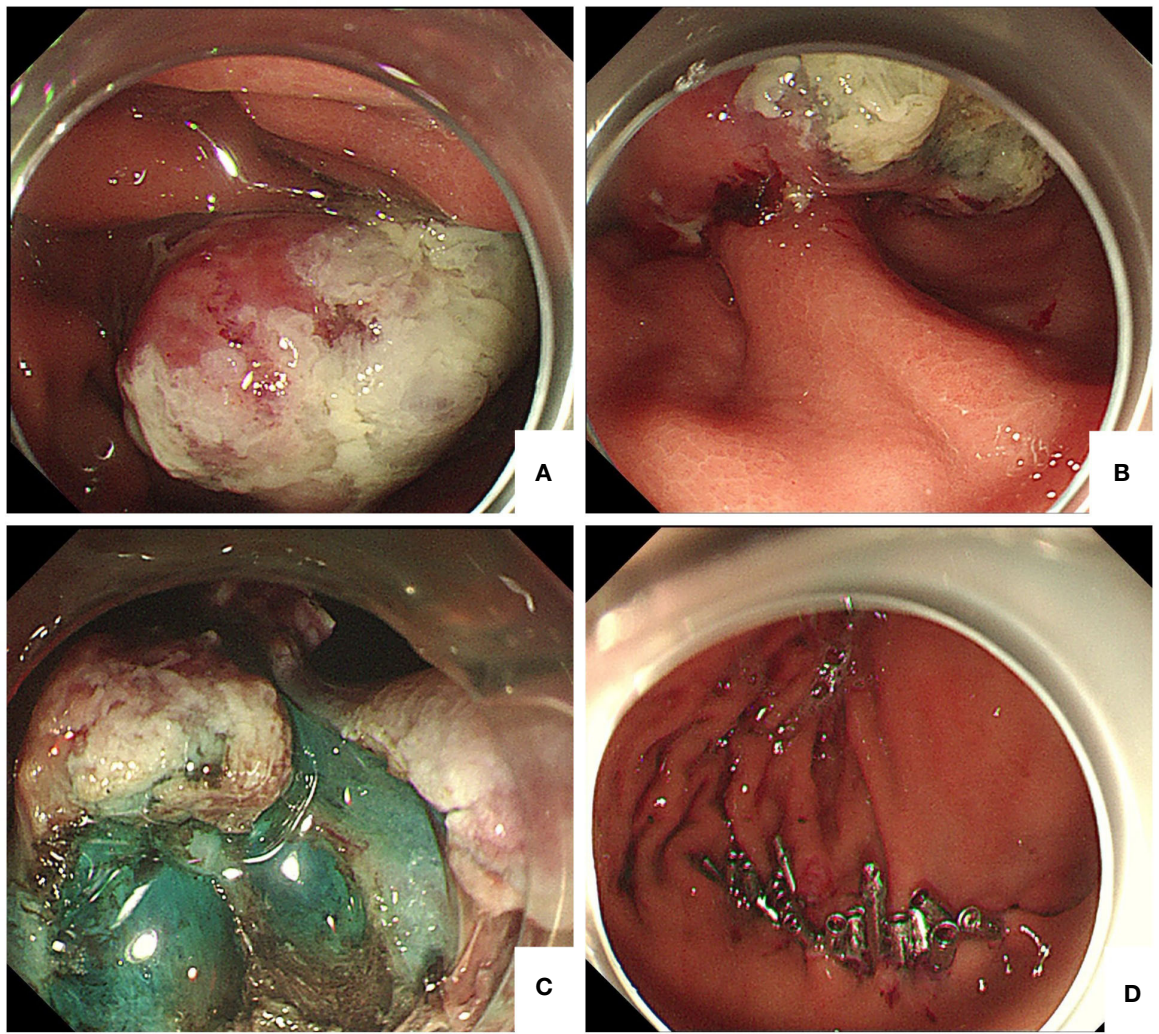
**(A)** Gastroduodenoscopy showing a 4 cm × 5 cm protuberant lesion on the greater curvature of the gastric body. **(B)** The base of the tumor was thickly pedicled. **(C)** After injection of methylene blue around the tumor, we found that the base of the tumor was closely adhered to the muscularis propria layer. **(D)** Titanium clip was used to completely close the wound.

**Figure 3:**
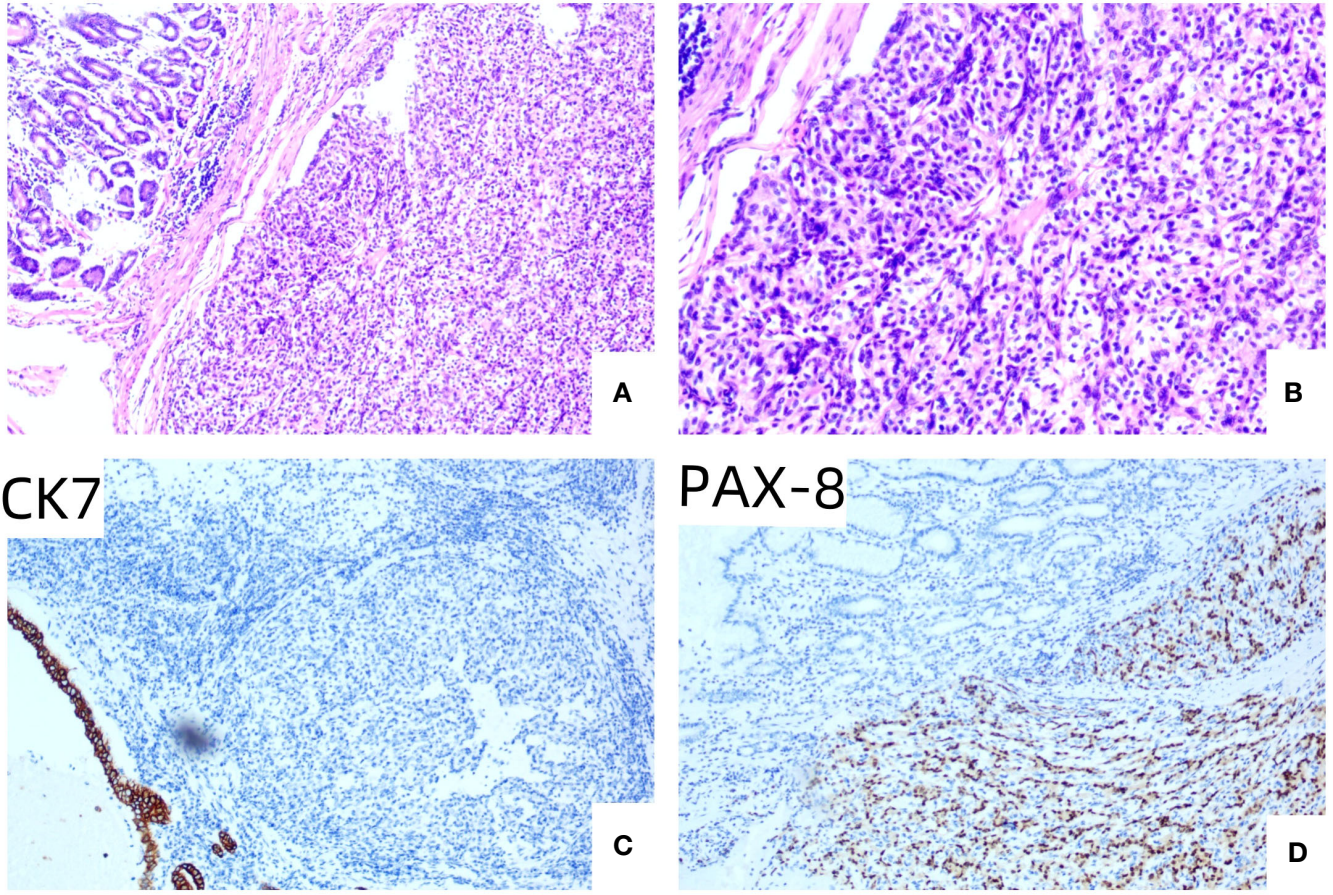
**(A)** HE × 100, the tubular structure in the upper left corner of the visual field was normal gastric mucosal gland, and the lamellar tumor cell nests were seen in the submucosa. **(B)** HE × 200, the cytoplasm of tumor cells is a translucent, low-grade nucleus surrounded by abundant capillaries. **(C)** IHC × 100, gastric mucosal epithelial CK7 staining was positive, and tumor cells were not expressed. **(D)** IHC × 100, PAX-8 staining was positive in tumor cells, but not in gastric mucosal epithelium.

An 82-year-old male patient presented at the hospital for “intermittent epigastric pain and melena for half a year”. The patient had undergone a gastroduodenoscopy examination in other hospitals 3 months ago, and the results only indicated a mass in the gastric body. Partial tissue was excised for pathological examination. Pathological results indicated that the mass was an inflammatory necrotic and granulation tissue. The patient underwent left radical nephrectomy for left renal clear cell carcinoma 12 years prior. He had been taking sorafenib orally after undergoing nephrectomy, but the medication was discontinued 2 months before he visited our hospital; for half a year, he experienced multiple epigastric pain and melena. After admission, the patient underwent an enhanced abdominal computed tomography (CT) scan, which suggested multiple metastases to the right kidney, pancreas, spleen, and stomach (**Figure 1**). A hematological examination showed HGB 93 g/L, and the patient was in a state of chronic blood loss. Before the operation, the patient’s gastric mass indicated large RCC metastasis, and we recommended that the patient undergo gastroduodenoscopy and pathological examination. We invited the gastrointestinal surgery, urology, and oncology departments for multidisciplinary discussions. The urology and oncology departments suggested that the patient could undergo immunosuppressive therapy combined with targeted drugs. Unfortunately, the patient and their family have abandoned further treatment, and the patient has not yet used any drugs (including sorafenib). Given the patient’s strong desire for endoscopic treatment, and after full communication with the patient and their family, gastroduodenoscopy and treatment under general anesthesia with tracheal intubation were performed. Gastroduodenoscopy revealed protuberant lesions measuring approximately 4 × 5 cm on the greater curvature of the gastric body, with an ulcerated surface and easy bleeding (**Figure 2A**). The base was thickly pedicled (**Figure 2B**), and endoscopic submucosal dissection (ESD) was planned. First, the area 0.5 cm away from the tumor was marked, and a 1:10,000 mixture of adrenaline and methylene blue was injected under the mucosa at the marked area. The lifting sign around the submucosa was good, and a dual knife was used to peel off the mucosa along the marked area. However, the dissection was close to the base of the tumor, the tumor adhered closely to the muscularis propria (**Figure 2C**), and ESD could not be continued; therefore, EFTR was performed. The dual knife was used for full-thickness resection of the gastric wall along 0.5 cm of the margins of the mass; with the assistance of titanium dental floss traction, the gastric tumor was fully resected. After observing that there is no bleeding at the site of gastric wall damage, the wound was sealed completely with a titanium clip (**Figure 2D**). After surgery, fasting, rehydration, anti-inflammatory drugs, acid suppression, gastrointestinal decompression, and other treatments were administered. After 8 days of close observation, the patient was discharged. Postoperative pathological results showed that the tumor of the greater curvature of the stomach was consistent with the metastasis of clear cell RCC (**Figures 3A, B**). The resected stomach specimen had a clear resection margin, and no tumor cells were found. Immunohistochemical results showed CAIX (+), CK broad-spectrum (+), Vimentin (+), PAX-8 (+), CD10 (+), CK7 (−), and Ki-67 (5%–10%) (**Figures 3C, D**), and finally a diagnosis of gastric metastasis of RCC. The patient was followed up for 6 months and did not have epigastric pain or melena.

## 3 Discussion

Gastric RCC metastasis may be a late recurrence event or a slow growth event; however, the 5-year survival rate of patients with resectable RCC metastases is higher (72.6%). Gastric metastasis of renal cancer cells is hematogenous and spreads to the fundus or body of the stomach and the submucosal gastric wall terminal artery (5). In the early stage, a slight bulge may be formed under the mucosa, which then grows gradually to the lumen side to form polyps or shows an ulcer appearance (6). Gastric metastasis also usually has non-specific manifestations of upper gastrointestinal bleeding, anemia, and abdominal pain. Although there is a long lag period from the initial diagnosis to the occurrence of gastric metastasis, approximately 6.7 years (range, 0–23 years) (7) on average, any patient with a history of RCC and gastrointestinal symptoms should be suspected of gastric metastasis (8). Complete resection of gastric metastases with bleeding is beneficial for improving the quality of life and overall survival time of patients. Some studies have shown that surgical resection is the best treatment option for gastric metastases (9). However, surgical resection, whether total or subtotal gastrectomy, requires a higher physical condition, greater trauma, and slower postoperative recovery, while EFTR is associated with less trauma and faster postoperative recovery. Therefore, EFTR is a more reasonable treatment option, and endoscopy plays an important role in the treatment of such patients (10). Few reports suggest the causes of gastric metastases after surgery for clear cell carcinoma of the kidney. Dabestani et al. concluded that complete resection of metastases can benefit overall survival and cancer-specific survival in these patients (11). Therefore, we recommend that gastroscopy should be performed in the follow-up of RCC after resection (12).

## 4 Conclusions

Our patient showed gastric metastasis 12 years after the initial RCC diagnosis; owing to the characteristics of RCC metastasis, early endoscopic diagnosis and treatment may be difficult. However, when the tumor develops to the point of resection, EFTR can retain gastric tissue and function to a greater extent than traditional surgical resection, and patients experience less pain. During palliative care, EFTR resection of isolated metastatic lesions in the stomach is important. This is because it effectively prevents complications, such as abdominal pain and gastrointestinal bleeding, and prevents a reduction in life expectancy due to complications. However, this requires more experience and technical skill from endoscopists.

## Data availability statement

The raw data supporting the conclusions of this article will be made available by the authors, without undue reservation.

## Ethics statement

The studies involving humans were approved by Ethics Committee of Shengli Oilfield Central Hospital. The studies were conducted in accordance with the local legislation and institutional requirements. The participants provided their written informed consent to participate in this study. Written informed consent was obtained from the individual(s) for the publication of any potentially identifiable images or data included in this article.

## Author contributions

XY: Writing – original draft. LL: Writing – review & editing. WW: Writing – review & editing. CL: Writing – review & editing. ZC: Writing – review & editing.
